# Quality of life in a broader perspective: Does ASCOT reflect the capability approach?

**DOI:** 10.1007/s11136-017-1756-5

**Published:** 2017-12-14

**Authors:** M. S. van Loon, K. M. van Leeuwen, R. W. J. G. Ostelo, J. E. Bosmans, G. A. M. Widdershoven

**Affiliations:** 1 Department of Medical Humanities, VU Medical Center Amsterdam, Amsterdam, The Netherlands; 2 Faculty of Earth and Life Sciences, Free University Amsterdam, Amsterdam, The Netherlands

**Keywords:** Quality of life, ASCOT, Capability approach, Economic evaluation

## Abstract

**Purpose:**

Economic evaluation of services and interventions in care services tends to focus on quality of life(QoL) based on health-related measures such as EQ5D, with a major focus on health and functioning. The Capability Approach (CA) provides an alternative framework for measuring QoL and challenges some of the conventional issues in the current practice of measurement of QoL. The Adult Social Care Outcomes Toolkit (ASCOT) aims to measure social care-related QoL in a broad sense. This article investigates whether and, if so, how the ASCOT addresses issues put on the agenda by the CA.

**Methods:**

Literature analysis concerning theoretical assumptions and arguments of CA and ASCOT.

**Results:**

The Capability Approach (CA) puts three issues on the agenda regarding QoL. First, the focus of evaluation should not be on functioning, but on freedom of choice. Second, evaluation should be critical about adaptive preferences, which entail that people lower expectations in situations of limited possibilities. Third, evaluation should not only address health, but also other domains of life. Our analysis shows that freedom of choice is reflected in the response option ‘as I want’ in the ASCOT questionnaire. The problem of adaptive preferences is countered in the ASCOT by developing a standard based on preferences of the general population. Third, the ASCOT contains several domains of life.

**Conclusions:**

We conclude that the CA and the ASCOT contribute to the discussion on QoL, and that the ASCOT operationalizes core assumptions of the CA, translating the issues raised by the CA in a practical way.

## Introduction

Societies are aging and the number of people living with a chronic disease is rising, resulting in a growing need for care services for older adults [1]. This increase in the demand for healthcare has led to an enormous increase in (health)care costs while the resources available are limited. Therefore, (health)care decision makers (e.g., policymakers and service providers) need to decide on how to spend these scarce resources best, for example, in decisions for national reimbursement and local commissioning. By doing so, decision makers aim to maximize the health benefits within their allocated budget [2]. Information to inform these decisions is often obtained from economic evaluations in which the costs and effects of two or more interventions are being compared. Such allocation decisions are generally taken using information from scientific studies in which the costs and effects of two or more interventions are being compared. Such studies are referred to as economic evaluations. Currently, economic evaluations focus on Quality Adjusted Life Years (QALYs) as the primary outcome [3, 4] in which both quality of life (QoL) and life gains are included.

The QALY measures length and QoL based on a health-related QoL measure such as the EuroQol-5D (EQ-5D). Yet, many interventions, treatments, or health care services lead to an improvement in outcomes other than health alone [2, 3, 5, 6; p. 1194, 5, 6]. For example, increasing mobility by providing a walker can influence someone’s ability to go outdoors, increasing a person’s feeling of being in control and autonomous, and enabling her to have a social life. In this way, many aspects of QoL can be influenced by an intervention aimed, not at changing health, but at supporting physical, psychological, and social functioning. QALYs are likely to underestimate the outcomes of such care interventions [7, 8], as many care receivers have chronic diseases in which improvements in health-related QoL such as operationalized in the EQ-5D are very unlikely, and often these domains are not specifically targeted by these services [2, 9]. Therefore, the standard of relevant outcomes needs to be broadened when evaluating interventions in social care, and we need to redefine what we consider as ‘value for money’ within this setting.

In care services for older adults, outcomes are now often evaluated on the basis of health-related QoL measures. The foundation of the QALY-framework, focusing on maximization of health, lies within the extra-welfarism approach in health economics [10, 11]. This approach might not always be in line with the goals and philosophy of these kinds of services. A promising alternative to implement in the evaluation of outcomes of services in long-term care for older adults can be the capability approach (CA), shifting the focus towards QoL in a broader sense [12].

The CA is a political, philosophical, and economical theory which presents a view on QoL, that goes beyond health. In the CA, well-being is the core concept. QoL, or, more generally, well-being, is assumed to consist of a variety of capabilities—we should evaluate what people are able to do and who they are able to be [13]. The focus is on enabling people to do the things they want to do. Since people are different and have diverse needs, the freedom to be able to live the life one wants and to do what one values is central in the CA. The CA has been implemented widely in developmental economy and proven to be a successful approach in this area [14]. In care services for older adults, the CA may provide a valuable framework for measuring QoL that shifts the focus from a narrow conception of health to a broader conception, and fits the lifeworld of receivers of this type of care [15–21]. For many care receivers, regaining health is not the most important goal, maintaining QoL, however is relevant to most of them [17, 19].

In recent years, different questionnaires with the purpose of evaluating outcomes of (health)care have been developed based on the CA, such as the OCAP-18, ICECAP, and the ASCOT [22]. In this article, we focus on the Adult Social Care Outcomes Toolkit (ASCOT), a measure that aims to measure QoL from a broader perspective than health alone which is partly based on the CA and now widely used in the United Kingdom for the evaluation of social care services [23, 24].

In this paper, we will address the following question: To what extent are issues raised by the CA concerning (the measurement of) QoL addressed by the ASCOT? We will first consider three main issues put on the agenda by the CA. Next, we will investigate whether and how the ASCOT addresses these issues. In the discussion, we will reflect on the findings and mention some topics that need further consideration.

## Methods

This paper is part of a larger project studying QoL in older adults, analyzing the philosophical background of ASCOT and applying the questionnaire to the Dutch context as an alternative to EQ-5D in economic evaluations. For this paper, theoretical literature on the CA was analyzed, focusing on the arguments for using this approach as an alternative for current ways of measuring QoL. Literature of the main authors of the CA was studied, and further literature was selected using a snow-ball method, collecting articles through references. Since much has been written about CA theory, with diverse goals, a selection was made on the basis of the purpose of this article, that is to investigate how the issues put on the agenda by the CA concerning measuring QoL are addressed in the ASCOT. We thus do not present a systematic overview of the CA literature, but specifically want to elaborate on the perspective of the CA on measuring QoL and its relation to the ASCOT. In our project the focus is specifically on older adults, but ASCOT and the CA can also be relevant for a broader population.

## Central issues of the CA

The CA has been developed within political philosophy, as a reaction to existing theories of justice, especially utilitarian welfarism and justice as equal distribution of resources. In utilitarian welfarism, ‘goodness’ is assessed in terms of subjective utility, or happiness. In Rawls’ Theory of Justice, the main claim is that justice requires an equal distribution of resources [25]. Sen, one of the founders of the CA argues in his Tanner Lectures ‘Equality of What?’ that neither utility, nor resources should be the focus of justice [26]. Rather, we should focus on people’s capability to achieve functionings [14]. According to Sen, welfarism is too much focused on happiness, disregarding people’s reflective valuations [27; p. 18]. Sen considers the focus in Rawls’ distributive justice on resources people receive, equally narrow. We should evaluate well-being, defined in the CA as what people can do with these resources. There are major differences in individual abilities to convert resources into capabilities. People with disabilities might require more resources (e.g., a wheelchair, or more money to buy certain tools) to attain a certain level of capability (e.g., mobility) than others. Thus for Sen, income (a common operationalization of resources in economics) is not automatically well-being, because people use resources differently. Therefore, measuring resources is too limited.

In the CA, capabilities are central. Capabilities are defined as ‘the alternative combinations of functionings the person can achieve, and from which he or she can choose one collection’ [13; p. 21]. Living is seen as combination of these ‘doings and beings’ and QoL is to be assessed in terms of the ‘capability to achieve valuable functionings’ [13; p. 21]. In the following sections, three main issues of the CA relevant to evaluation of care services will be elaborated. The issues regard the importance of freedom and choice, the need to be critical of adaptive preferences, which entail that people lower their expectations, because they adapt to deteriorated circumstances, and the need to take into account several relevant domains of life.

### Freedom and choice

Sen emphasizes the relevance of freedom and choice [14]. Freedom is valuable because ‘it gives us more opportunity to pursue our objectives.’ Moreover, the ‘*process* of choice itself’ is important [14; p. 228]. People should be able to live the life they want to live, and have the ability to choose certain functionings. Starving and fasting imply the same functioning—not eating—but the person who fasts still has the capability to eat, whereas the starving person has not [14]. A central idea of the CA is that having certain capabilities is fundamental for QoL and that, by protecting and restoring peoples’ capabilities, well-being will increase [13, 14]. Care services may, for example, protect and restore people’s QoL by providing support in everyday life activities which enables them to choose/realize certain functionings (e.g., physical therapy can support mobility, and in this way improves the capabilities of a person by providing her more options, or day-care activities can enable people to meet others, and provides more options for social relationships).

The CA takes into account human diversity. The focus on freedom and choice in the CA hosts diversity of people with different ‘life objectives,’ backgrounds, opportunities, and conversion factors. The CA is a liberal framework which tries to avoid paternalism by focusing on freedom and choice, rather than stating certain functionings as important for everyone [28, 29]. QoL is different for everyone [29]. Capability, and not functioning, is seen as the correct political goal by capability theorists [28, 30; p. 101]. Physical handicaps can mean that people require different kinds of services to achieve a certain capability [30]. Also, if the same capabilities are present, people may choose different functionings, related to what they find valuable in living their lives [14].

People have different abilities to convert resources into capabilities. A person unable to walk requires more resources to be able to move in an environment, to compensate for this disadvantage. Therefore, according to the CA, in evaluations of well-being outcomes, we should measure whether people are able to do what they would like to be able to do instead of the services they receive or how happy they are [30]. Do they have options to choose from functionings they value? If not, we should create the circumstances to enable them to choose valuable things. In her version of the CA, Nussbaum [31] argues that society should enable people to fulfill certain basic capabilities such as being able to be nourished and educated; we should evaluate if the conditions for individual capabilities are met and protect and restore individual capabilities.

### Overcoming adaptive preferences

Sen [14, 24] criticizes welfarism and argues that utility overemphasizes ‘mental and emotional responses to commodities (resources) and characteristics of commodities and not enough on what they enable you to do’ [32; p. 51]. The CA argues against measuring so-called ‘adaptive preferences’ [31; p. 34]. Being in a certain situation can influence a person’s experienced happiness and expectations of what is possible. Patients with severe medical conditions, for example, often lower their expectations of what life can bring [14, 30]. Even in a state of severe physical distress, patients may still consider their health as fine according to their lowered standards. When people are isolated, they can accommodate and feel that there is no need for more social contact. However, this does not mean that their situation cannot or should not be improved; care services are still needed to support and foster their QoL. Measuring the level of well-being in terms of utility might not grasp unjust circumstances, since expectations may have been adapted to the current, disadvantaged situation [14, 27, 32]. Therefore, an evaluation that focuses only on subjective mental metrics is insufficient without considering whether that matches with what a neutral observer would perceive as their objective circumstances. An external standard of well-being is needed in order to judge whether a situation requires improvement [14, 28]. Such an external standard may, however, be at odds with the emphasis on personal choice discussed in the previous section. We will go into this tension in the “Discussion.”

### Multiple domains

The CA argues that evaluations of well-being should take into account multiple domains. According to Sen [27] we have to select certain relevant capabilities/functionings dependent on the setting, and attach weights, in order to make a QoL evaluation (p. 25). Nussbaum [31] constructed a list of Central Human Capabilities (CHCs), incorporating the moral entitlements of every human being. According to Nussbaum, people need a certain threshold level of CHCs, to lead a dignified human life and to flourish [31]. Nussbaum distinguishes ten CHCs: life; bodily health; bodily integrity; senses, imagination, thought; emotions; practical reason; affiliations; other species; play; control over one’s environment (and being able to live one’s own life) [31; pp. 41–42]. Sen is critical of making a universal list of capabilities, because, he argues, different sets will be relevant to different groups and in distinct settings [33; pp. 157–160]. Moreover, according to him, making a list disregards the liberal nature of the CA, since what contributes to ‘quality of life’ is determined by others than the people themselves.

Sen does not provide concrete suggestions for relevant capabilities, but states that for each context ‘some democratic process and public reason should be involved’ [25, 33; p. 356]. Democratic processes of reasoning are crucial to select relevant capabilities, and decide which capabilities we have ‘reason to value,’ because ‘what we may value is very diverse’ [14; pp. 231–233]. In a pluralistic society, people disagree about values, have different ideas about which aspects of life contribute to QoL. His view is that through a process of reasoning, values and ideas about what QoL entails, can be made explicit and be made the object of deliberation. We should investigate for each particular context which capabilities are relevant.

Capabilities can be specified by empirical research as argued by several authors [29, 32, 34], either to construct for each context an index of relevant capabilities (Sen’s version), or to concretize the general list of Nussbaum’s CHC in a certain context. Qualitative techniques can be used for the selection of functionings and for determining their importance [34]. When QoL is defined in terms of a variety of domains, weights should be attached to determine the importance of each domain in a specific context. This attachment of weights to different domains has to be ‘done in terms of *explicit* evaluations, drawing on the prevailing values in a given society’ [34; p. 25]. There is discussion about who should decide what values are and how weighs should be attached [8, 30, 35, 36]. The CA does not provide straightforward answers to these questions and the theory is operationalized in different ways [29, 37, 38].

## The ASCOT

The ASCOT was developed for measuring QoL, focusing on the goals of social care services. Social care services aim to provide support in basic functionings, such as nourishment and personal hygiene [39], and are, therefore, concerned with reducing the effect of impairment on people’s daily life [40; p. 1]. During later development phases of ASCOT, the notion of capability was introduced, referring to recent policies aspiring to ‘broaden[ing] opportunities for people with disabilities and developing ‘independence’, ‘choice’ and ‘control’’ [39; p. 1]. The presupposition behind the reference to capabilities is that service users are expected to value an increase of freedom and flexibility as outcome of services [39]. The ASCOT connects to capability theory and to more general societal developments by focusing on what social care receivers are able *to do*, on their capabilities, rather than on impairment and limitations. Since people have different needs (and wants), the produced value of a service varies per person [41; p. 3]. The ASCOT aims to measure these divergent outcomes [23, 39, 41].

**Fig. 1:**
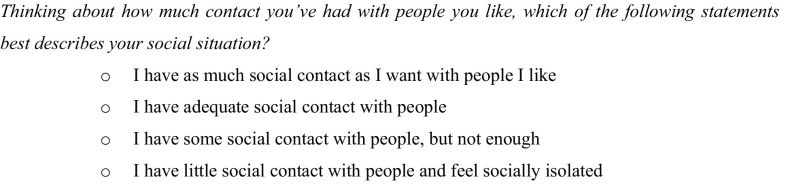
Exemplary question of ASCOT

The ASCOT toolbox consists of several instruments and can be used for evaluation of a wide range of services and settings. In this paper, we focus on the 4-level self-completion tool (SCT4) questionnaire^1^ [39, 40]. ASCOT SCT4 assesses 8 different domains of QoL: (1) control over daily life, (2) personal cleanliness and comfort, (3) food and drink, (4) personal safety, (5) social participation and involvement, (6) occupation, (7) accommodation cleanliness and comfort, and (8) dignity. All domains have 4 response options; the first response option represents the ideal situation and the last one represents the worst imaginable state. An exemplary question for the ‘social participation and involvement’ domain is shown in Fig. 1.

### Freedom and choice

In the ASCOT, it is assumed that some basic functionings are important for everyone: ‘although preferences might differ, it is hard to imagine that any person is not better off if they are fed, clothed and sheltered than not’ [40; p. 8]. Preferences for more complex functionings, however, can differ more substantially between people. Social contact, for example, can be less important for some people than for other people [40]. Therefore, especially in complex functionings, the *potential* to function is important, which indicates that capability should be addressed when measuring well-being.

In the SCT4 version, the notion of freedom of choice as a crucial aspect of capabilities is reflected in the response option ‘as I want’ [39]. This response option of SCT4 represents an ideal situation of full capability indicating that people are not restricted in the level they want to achieve, and the three lower options represent more basic functionings, indicating care needs [24, 39]. Moreover, a specific domain is included which focuses on having freedom to choose important issues in life, namely ‘control over daily life’ [24, 39]. Netten et al. [24] argue that it is important to measure ‘the full range from the very fundamental level where functioning levels are so low they could lead to mental and physical health implications, through to ‘capability’ states, in which people have real choice’ [24].

### Overcoming adaptive preferences

In the ASCOT, the possibility of adaptive preferences is addressed in three ways. First, the ASCOT is based on a standard of basic functionings, the domains in the questionnaire. Some functioning states can be judged to be unacceptable by society (e.g., malnourishment), and a standard facilitates the argument that certain functioning states are too low [28, 39; p. 2]. Second, in completing the ASCOT respondents are encouraged to reflect upon their preferences by including ‘as I want’ response option. Is their situation ideal, or is there room for improvement? Third, for the calculation of a weighted total score the valuation of the different domains is based on the judgment (preferences) of situations by people from the broader society (see “Multiple domains”). Although the ASCOT does not take adaptive preferences for granted, they are not regarded as intrinsically wrong [24]. Coping is considered positive, since social care can support people’s adaptation to changed circumstances, for example, by helping people to reduce the effects of impairment on people’s daily lives [24]. However, we should not conclude, based on measuring adapted preferences, that a person’s situation cannot be improved.

### Multiple domains

Domains in the ASCOT have been taken from a previous project in which relevant domains of well-being in older adults were obtained from discussion with experts in the field, focusing on the question: what are the aims of social care services? [40] Additionally, the team drew on a contemporaneous large-scale qualitative project that examined how social care users define social care outcomes, using focus groups and interviews. The domains identified in this study fed into the final specification of the ASCOT domains [24]. The domains in the ASCOT are broader than current health-related QoL measures, for example, the EQ-5D which measures health and mobility as important domains. Domains in the ASCOT like (1) control over daily life, (4) personal safety, (5) social participation and involvement, and (8) dignity are concepts that go beyond health and mobility.

In the ASCOT, for each level within a domain, set weights have been estimated. The weights for domains were developed through various studies, and the actual weights were developed by Netten et al. [24, 41]. Techniques used to establish preference weights for the eight domains are best–worst scaling (BWS) methods in combination with time-trade-off (TTO) methods [24] with members of the general population and social care service users. The analysis showed that there are ‘no substantive’ differences in preferences between service users and the general population [24]. The final model is based on preferences of ‘1000 members of the general population’ [24].

## Discussion

In this section, we will compare the CA and the ASCOT on the three issues mentioned before and mention some aspects which require further investigation. The main recommendations that we distilled from theoretical analyses of the CA [13, 14, 28, 29] are (1) we should be aware of diversity in people and thereby the diversity regarding personal freedom and choice; (2) we should be aware of the fact that people tend to adapt to their situation and (3) we should take into account multiple domains when measuring QoL, specified for different contexts and target groups. In this article, we focus on a comparison with ASCOT, but these recommendations are relevant for other capability measures too.

### Freedom and choice

The CA underlines that people should have the freedom to choose between various functionings [14, 29]. Operationalizing freedom is a central issue in capability literature debate on this topic [22, 29]. In the ASCOT, the importance of freedom and choice is taken into consideration by including ‘as I want’ in the response options, as well as adding a ‘control’ domain [24, 27]. These elements in the ASCOT reflect the notions of freedom and choice in the CA. Yet, some topics need further consideration. In the first place, it may be discussed whether ‘as I want’ properly addresses the issue of freedom. Does the clause ‘as I want’ motivate the respondent to actually reflect and value the current situation, or does it merely lead to an indication of being content? Empirical clinimetrical research [42] indicates that there are significant differences in autonomy and control (as measured by the CASP subscale—a scale measuring control and autonomy) between the top two options of each ASCOT domain (‘as much as I want’ versus ‘adequate’). Equally there are significant differences in CASP subscale scores between each level of the control domain. This would suggest that the difference in wording between the top two options for each ASCOT domain is reflecting control and autonomy, at least as it is described by the CASP subscale. Other research indicates that capabilities can indeed be self-reported; participants explained to understand the capability concept as ‘capacity’ and something they ‘could do.’ These empirical studies suggest including ‘as I want’ could encourage respondents to reflect on their capabilities [43; p. 119].

Within each domain, respondents have the freedom to determine their own choices, by valuing their own situation by judging the situations within a domain. The domains are always the same in ASCOT, restricting people in determining what QoL entails. Some domains might be less relevant for some service users (they might not care for social contact), and those important to some may not be included in the current list (e.g., spirituality). However, for most of the domains, one can hardly doubt the relevance. It is difficult to imagine that persons are not better off if they are fed, clothed, and sheltered than not.

### Overcoming adaptive preferences

In current economic evaluations of health care, usually indirect utility instruments such as the EQ-5D are used [3, 44]. Participants indicate their health status using a questionnaire consisting of several QoL domains and a pre-specified set of weights is used to value each health status, based on preferences of the general population [44]. In the ASCOT, taking into account adaptive preferences is an important topic [24, 39]. The instrument aims to measure QoL in reference to a standard. In order to determine the standard, the results of social care professionals (for determining which domains are important) and the general public (for determining weights) are used. In this respect, the ASCOT follows the line of the CA [39]. Yet, making a standard list is problematic, as it assumes that QoL is the same for every person, and consists of specific capabilities regardless of individual values [28]. This leads to a tension in the CA approach; the idea is to be open for different conceptions of QoL but in order to be able to compare well-being outcomes between various persons, fixed domains are needed. An alternative to account for diversity might be measurement scales where respondents can define their own outcome domains such as Schedule for the Evaluation of Individual Quality of Life (SEIQol) [45] Although this might account for diversity in people’s conception of QoL, adaptation complicates interpersonal comparison, hindering its use in economic evaluations.

As the ASCOT SCT-4 is a self-completion questionnaire, respondents evaluate their *own* situation, based on individual reflection. This approach has the advantage that respondents make their own judgment, and that other people do not make this judgment for them. Yet, this also gives room for individual interpretation, which may be influenced by adaptation to the situation [12, 14]. The answer options are broadly defined, and, as mentioned before, the clause ‘as I want’ can be interpreted in various ways. Thus, adaptive preferences are not fully excluded. This might not be problematic per se. As argued by various authors [30, 46, 47], it is good that people adapt to their situation. Adapting means people cope with deteriorating conditions [48]. The ASCOT team suggests that in order to judge whether adaptation is problematic or not, a more ‘objective’ expert could judge a person’s capability set [24]. This could be an experienced professional, a proxy, family, or informal caregivers. However, in the CA theory it is assumed that individuals should have the freedom to choose between functionings [14]. The issue of measurement of adaptation is currently debated within the capability literature, since it is a recurring theme in operationalizing CA for measurement purposes [28, 47, 49, 50]. We believe that a possible solution might be an intersubjective approach, combining a subjective element (the respondents own evaluation) with a more objective list (including set domains, developed in empirical research, and evaluation by other people). For example, in the ASCOT care home version, data are triangulated obtained from trained observers, residents, and staff/family. In this way, multiple perspectives are combined. Such an approach is however more time consuming (and costly).

### Multiple domains

In the ASCOT, QoL consists of multiple domains, in line with the CA. The domains of the ASCOT are also congruent with both the examples mentioned by Sen, and the list of central human capabilities proposed by Nussbaum [14, 31]. The latter does, however, entail wider elements which are not addressed in the ASCOT. It may be further discussed whether these capabilities should be the object of social care interventions. QoL may encompass domains which are currently not covered by the ASCOT, or domains specific to certain contexts or countries. Systematic reviews in specific target groups to analyze what QoL entails and what should be measured for these groups are recommended. This might be addressed by organizing processes of deliberation on QoL, involving stakeholders, especially older people or other care receivers themselves, in line with Sen’s idea [14].

In these empirical processes of deliberation, supported by qualitative research, for example, in focus groups, well-known problems of democratic processes, such as the power of the majority should be countered [29]. It is possible that people disagree on relevant domains [38]. The majority can outweigh the opinions of minorities on including certain capabilities [29]. In this way, the majority may decide what the good life is, not based on the quality of arguments, but on quantity. It is important to prevent ‘ethics by opinion poll’ [51]. Thus, including stakeholders requires careful procedures to counteract a system of voting and create conditions for an open dialogue between various parties involved. In empirical ethics, qualitative methods have been developed to include the perspectives of stakeholders [52, 53]. These methods entail in-depth interviews with stakeholders, and further exploration of the results of the analysis of these interviews in focus groups, both homogeneous groups, in which for instance healthcare professionals, patients, and family discuss issues raised in the interviews among themselves, and heterogeneous focus groups, in which various stakeholders exchange experiences and views to better understand each other’s perspective and jointly develop new shared insights. Such methods might be helpful to further contextualize QoL measurements.

## Conclusion

The CA is relevant for the evaluation of care services, shifting the focus from health-related QoL to a broader definition. The ASCOT can be regarded as an example of a broad measure of QoL, evaluating the outcomes of care services on more domains than health.

The CA puts three central issues on the agenda. The first is the need to focus on freedom and choice, and to pay attention to diversity in what people need, want, and can do with services. The second is the need to be critical about adaptive preferences, since poor conditions can give rise to lowered expectations. The third is the need to take into account that QoL consists of multiple domains. In general, these issues are addressed in the ASCOT. Thus, it is a promising instrument to evaluate long-term care services from the perspective of human capabilities.

Some aspects require further attention in future research. The first is the question whether the option ‘as I want’ and the domain of control over daily life in the ASCOT are adequate operationalizations of the notion of freedom in the CA. Does the sentence ‘as I want’ stimulate reflection on values in the way in which this is meant by the CA? In developing QoL questionnaires, it is important that instruments measure several domains and this measurement about QoL states is partly subjective (self-reporting, ‘open’ response options) and partly objective (fixed domains and weighting). More attention for diversity in questionnaires seems warranted to account for differences in personal needs and wishes of respondents. Furthermore, the influence of this personal weighting in the context of cost-effectiveness should be explored. The second is related to adaptation. Although one should be critical about adaptation to situations which can be improved, adaptation may not be fully prevented, and even regarded as positive in a situation in which one’s physical abilities are diminishing. How to determine whether and when adaptation is acceptable, and even desirable? Here, an intersubjective approach might be useful, going beyond subjective and objective measures. Finally, the determination of domains and levels of capabilities requires attention. How to involve stakeholders in processes of deliberation, and organize democratic ways of answering the question which aspects of QoL are relevant in present society, and should be supported by care services? Developers of questionnaires should be aware of the needs and wishes of specific groups, and design methods for involving them in dialogical way. These issues for further research are not easily addressed. Yet, the CA and the ASCOT contribute to the discussion on QoL by raising awareness of the importance of these topics and suggesting pathways for further investigation.

## Footnotes

1 The full ASCOT SCT-4 questionnaire can be requested at http://www.pssru.ac.uk/ascot/instruments.php.
